# Severity of illness scores in the pediatric intensive care unit: a practical guide

**DOI:** 10.62675/2965-2774.20240205-en

**Published:** 2024-10-11

**Authors:** María del Pilar Arias López, Arnaldo Prata-Barbosa, Fernanda Lima-Setta

**Affiliations:** 1 Hospital de Niños Ricardo Gutierrez Pediatric Intensive Care Unit Buenos Aires Argentina Pediatric Intensive Care Unit, Hospital de Niños Ricardo Gutierrez - Buenos Aires, Argentina.; 2 Sociedad Argentina de Terapia Intensiva SATI-Q Program Buenos Aires Argentina SATI-Q Program, Sociedad Argentina de Terapia Intensiva - Buenos Aires, Argentina.; 3 Instituto D’Or de Pesquisa e Ensino Department of Pediatrics Rio de Janeiro RJ Brazil Department of Pediatrics, Instituto D’Or de Pesquisa e Ensino - Rio de Janeiro (RJ), Brazil.

Pediatric intensive care units (ICUs) concentrate specialized human resources and advanced technology that can improve the prognosis of critically ill children. However, while the pediatric ICU mortality rate has decreased to approximately 2% in high-resource countries, it remains close to 4% in others, such as those in Latin America. Therefore, there is a need to assess pediatric ICU performance and implement improvement strategies.^(1,2)^

The standardized mortality rate, calculated as the ratio of observed deaths to expected deaths, is a key indicator for evaluating the performance of a pediatric ICU. Although assessing observed mortality is easy, determining the expected mortality is challenging. Severity-of-illness scores (SIS) have emerged as tools that allow estimating the risk of death at the time of admission. Additionally, organ dysfunction (OD) scores have been developed to describe the clinical evolution of pediatric ICU patients.

Severity-of-illness scores and OD scores can be used to evaluate institutional performance for the purposes of internal and external benchmarking, guiding quality improvement initiatives, treatment allocation, and research.^(3)^ This viewpoint aims to provide an updated practical guide to enhance their use in pediatric ICU settings.

## UNDERSTANDING SEVERITY-OF-ILLNESS SCORES

The severity of illness is generally assessed based on a variety of demographic, clinical, physiological and laboratory variables. The SIS used in pediatric ICUs are based on the assumption that there is a predictable relationship between the severity of illness at the time of admission and the risk of death in the pediatric ICU. Essentially, they are mathematical models derived by applying stepwise logistic regression to an observational cohort, in which a specific value is assigned to each mortality-predictive variable, resulting in final odds transformed into the probability of death. Predictive variables can include physiological alterations (such as blood pressure, heart rate, and laboratory values), the need for interventions (such as mechanical ventilation and inotropes), the patient's underlying condition (such as malignant disease) and emergency admission to the pediatric ICU, among others. The performance of the resulting model is then assessed on the basis of its overall discrimination and calibration. If both measures are adequate, before the model is used in daily practice, it must be validated in a separate sample of patients from the same population (internal validation).

Considering the heterogeneity of the populations in pediatric ICUs (age, case mix), prognostic scores are established independently of the diagnosis to increase the objectivity of outcome assessment between institutions.

Description and quantification of OD during hospitalization in the pediatric ICU are important. Greater physiological instability upon admission, secondary to more severe illness, is correlated with a greater risk of developing multiple and sequential organ failure, which increases the mortality rate. Organ dysfunction scores have been developed and validated to enable description of the clinical course and the severity of illness during a stay in the pediatric ICU rather than merely to predict mortality.

The two most commonly used scores for mortality prediction are the latest versions of the pediatric risk of mortality (PRISM) and the pediatric index of mortality (PIM).^(4,5)^ The pediatric logistic organ dysfunction score (PELOD) and pediatric sequential organ failure assessment score (pSOFA) are used to assess multiorgan dysfunction.^(6,7)^ These scores, their construction guidelines and their limitations are listed in table 1.

**Table 1:** Mortality and severity-of-illness scores in the pediatric intensive care unit

**PIM3**	Developed in Australia, New Zealand, UK, Ireland^(5)^ **Previous versions:** PIM2 (2003); PIM (1997) **Variables (collected within the first hour of pediatric ICU admission):** pupillary reaction | Elective or emergency admission | mechanical ventilation at any time during the first hour | base excess | SBP | PaO_2_ and FiO_2_ | postoperative of cardiac surgery with bypass | postoperative of cardiac procedure without bypass | postoperative of noncardiac surgery | reason for pediatric ICU admission (very-high-risk diagnosis: cardiac arrest preceding ICU admission, severe combined immune deficiency, leukemia or lymphoma after first induction, bone marrow transplant recipient, liver failure), (high-risk diagnosis: spontaneous cerebral hemorrhage, cardiomyopathy or myocarditis, hypoplastic left heart syndrome, neurodegenerative disorder, necrotizing enterocolitis), or (low-risk diagnosis: asthma, bronchiolitis, croup, obstructive sleep apnea, diabetic ketoacidosis, seizure disorder). **Calculation:** Logit(P) = − 1.7928 + (3.8233×pupillary reaction − (0.5378×elective admission) + (0.9763× mechanical ventilation) + (0.0671×(absolute base excess)) − (0.0431×SBP) + (0.1716×(SBP×SBP/1,000)) + (0.4214×(100×FiO_2_/PaO_2_)) − (1.2246×postoperative of cardiac surgery with bypass) − (0.8762×postoperative of cardiac procedure without bypass) − (1.5164×postoperative of noncardiac surgery) + (1.6225×very-high-risk diagnosis) + (1.0725×high-risk diagnosis) − (2.1766×low-risk diagnosis) **Probability of death:** P = exp(logit(P))÷(1 + exp(logit(P)) **Special considerations:** Measures pediatric ICU mortality. The probability of death is not interpretable at the patient level and the score cannot be used for an individual diagnostic or therapeutic decision in managing the patient.
**PRISM IV**	Developed in the USA^(4)^ **Previous versions:** PRISM III (1996); PRISM II (1988); PSI (1984) Variables (collected within the 2 hours before admission and the first 4 hours of pediatric ICU admission for laboratory data, and the first 4 hours of pediatric ICU care for other physiologic variables)*: age | admission source (another hospital, inpatient unit, emergency department) | CPR within 24 hours before pediatric ICU admission | cancer (acute or chronic) | low-risk systems of primary dysfunction (endocrine, hematologic, musculoskeletal, and renal systems) | temperature | neurologic score: pupillary reactivity and mental status (Glasgow coma scale) | non-neurologic score: heart rate, systolic blood pressure, temperature, arterial PaO_2_, pH or total CO_2_, PaCO_2_, total bicarbonate, glucose, potassium, blood urea nitrogen, creatinine, white blood cell count, platelet count, prothrombin or partial thromboplastin time. *If admission was for cardiac surgery or interventional (nondiagnostic) cardiac catheterization, perform PRISM IV within 4 hours after surgery or intervention if the patient is between 12 hours and 10 days old or is between 11 and 90 days old and within < 48 hours of pediatric ICU admission. **Calculation:** Logit(P) = − 5.776 + (1.311×age 0-14 days) OR (0.968×age 14 days-1 month) OR (0.357×age 1-12 months) + (1.012×admission another hospital) OR (1.626×admission inpatient unit) OR (0.693×admission emergency) + (1.082×CPR 24 hours before admission) + (0.766×cancer) – (1.697×low-risk systems) + (0.197×neurologic score) + (0.163×non-neurologic score) **Probability of death:** P = exp(logit(P))÷(1 + exp(logit(P)) **Special considerations:** measures hospital mortality, which may affect comparisons when evaluating outcomes with other scores. The probability of death is not interpretable at the patient level and the score cannot be used for an individual diagnostic or therapeutic decision in managing the patient
**PELOD2**	Developed in France and Belgium^(6)^ **Previous versions:** PELOD (1999) **Variables (collected within the first 24 hours and repeated daily):** cardiovascular (lactatemia and mean arterial pressure) | respiratory (PaO_2_/FiO_2_, PaCO_2_ and invasive mechanical ventilation) | neurologic (Glasgow coma scale and pupillary reaction) | hematologic (white blood cell and platelet count) | renal (serum creatinine) **Calculation:** PELOD-2 score = neurologic dysfunction [(Glasgow coma score: 0, 1 or 4 points) + (pupillary reaction: 0 or 5 points)] + cardiovascular dysfunction [(lactatemia: 0, 1 or 4 points) + (mean arterial pressure: 0, 2, 3 or 6 points)] + renal dysfunction (creatinine: 0 or 2 points) + respiratory dysfunction [(PaO_2_/FiO_2_: 0 or 2 points) + (PaCO_2_: 0, 1 or 3 points) + (invasive ventilation: 0 or 3 points)] + hematologic dysfunction [(white blood cell count: 0 or 2 points) + (platelets: 0, 1 or 2 points)] Logit(P) = − 6.61 + (0.47×PELOD-2 score) **Probability of death:** P = exp(logit(P))÷(1 + exp(logit(P)) **Special considerations:** the data recorded by the score are influenced by the treatment. The score cannot differentiate between incorrect treatment and the severity of the illness. Variables are measured only if the patient's clinical condition justifies it, assuming normality otherwise. Assessing neurological status is difficult because of the need for sedation in critically ill patients, so the presedation status is assumed.
**pSOFA**	Developed in the USA from the adult SOFA score using age-adjusted criteria^(7)^ **Previous versions:** SOFA (1996, adult version) and Sepsis-3 (2016, adult version) **Variables (collected daily during pediatric ICU stay):** respiratory (PaO_2_/FiO_2_ OR SpO_2_/FiO_2_) | cardiovascular (mean arterial pressure OR vasoactive infusion) | hepatic (serum bilirubin level) | coagulation (platelet count) | renal (serum creatinine) | neurologic (Glasgow coma scale) **Calculation:** pSOFA score = respiratory [(PaO_2_/FiO_2_ OR SpO_2_/FiO_2_) + coagulation (platelet count) + hepatic (bilirubin) + cardiovascular (mean arterial pressure by age group or vasoactive infusion) + neurologic (Glasgow coma score) + renal (creatinine) Daily pSOFA = the score for each of the six systems ranges from 0 to 4 points, total score ranges from 0 to 24 points **Probability of death:** the pSOFA score was not validated to calculate the risk of death. **Special considerations:** the data recorded by the score are influenced by the treatment. The score cannot differentiate between incorrect treatment and the severity of the illness. Variables are measured only if the patient's clinical condition justifies it, assuming normality otherwise. Assessing neurological status is difficult because of the need for sedation in critically ill patients, so the presedation status is assumed.

PIM - Pediatric Index of Mortality; ICU - intensive care unit; SBP - systolic blood pressure; PaO_2_ - partial pressure of oxygen; FiO_2_ - fraction of inspired oxygen; PRISM - Pediatric Risk of Mortality; CPR - cardiopulmonary resuscitation; CO_2_ - carbon dioxide; PaCO_2_ - partial pressure of carbon dioxide; PELOD - Pediatric Logistic Organ Disfunction Score; SOFA - Sequential Organ Failure Assessment.

## KEY POINTS FOR CLINICAL PRACTICE

Selection of an SIS or OD score requires consideration of a variety of factors, such as ensuring that it has been validated locally, that it is updated, and that the personnel responsible for data entry are trained in the score's construction guidelines to ensure that the data are of high quality.

Severity scores are typically developed in high-income countries. If an SIS is intended to be used in populations other than that for which it was developed, it must be externally validated by performing discrimination and calibration tests. Discrimination is a measure of the ability of a score to assign lower probabilities of death to patients who will live and higher probabilities of death to those who will die. It is evaluated by calculating the area under the Receiver Operating Characteristic (ROC) curve. An area under the ROC curve equal to 0.50 means that the score is not more discriminating than chance, an area between 0.70 and 0.79 is considered adequate, one between 0.80 and 0.89 is considered good, and an area > 0.90 is considered excellent.

Calibration is a measure of how well the predicted mortality matches the observed mortality by severity level at the time of admission to pediatric ICU. Specific statistical tests (e.g., Hosmer–Lemeshow and calibration belts) are used to compare observed and predicted deaths by severity range.^(8-10)^ When the calibration of a score in a population is good, the number of observed deaths in each severity decile is similar to the number of deaths predicted by the score in the same risk range. External validation of mortality prediction scores typically indicates poor calibration. In three large recent studies conducted in Latin America, PIM2 and PIM3 had good discrimination but poor calibration in several patient subgroups.^(11-13)^ This finding should be interpreted with caution. Although it may be due to the different performances of local pediatric ICUs, the result may have been influenced by regional or national variations in the organization of care and different patient–case combinations. If so, it would be useful to validate the score in a local representative sample and consider the obtained standardized mortality rate to be 1 (one) for that population.

As critical care treatments improve, outcomes for the same severity of illness can change. Therefore, an SIS needs to be updated by recalculating the coefficient of each variable or incorporating new variables. An accurate assessment of pediatric ICU performance requires the use of updated scores.

Data quality is an important consideration when developing and using a score. Errors in data recording and missing data can skew the results of even the most sophisticated score. Therefore, it is crucial to understand the construction rules of the scores that will be used in the pediatric ICU.

Finally, although mortality probabilities for individual patients can be calculated, they should not guide decisions regarding life support limitations.

## CONCLUSION

Pediatric intensive care units should use updated and preferably locally validated severity-of-illness scores to measure their performance. Also, measuring the degree of organ dysfunction at the time of admission and during a stay in the pediatric intensive care unit enables evaluation of a patient's progress and can be used as inclusion and exclusion criteria for treatment protocols. Implementing procedures to ensure data quality, such as real-time data collection by adequately trained personnel, is very important to ensure that predictions are reliable.
