# Supplementary material for: Severity of illness scores in the pediatric intensive care unit: a practical guide
Source: Crit Care Sci. 2024 Oct 11;36:e20240205en. doi: 10.62675/2965-2774.20240205-en (PMC11554295; doi:10.62675/2965-2774.20240205-en)
Supplement: Translation

# Translation: Escores de gravidade da doença na unidade de terapia intensiva pediátrica: um guia prático

**DOI:** 10.62675/2965-2774.20240205-pt

**Authors:** María del Pilar Arias López, Arnaldo Prata-Barbosa, Fernanda Lima-Setta

**Affiliations:** 1 Buenos Aires Argentina Unidade de Terapia Intensiva Pediátrica, Hospital de Niños Ricardo Gutierrez - Buenos Aires, Argentina.; 2 Buenos Aires Argentina Programa SATI-Q, Sociedad Argentina de Terapia Intensiva - Buenos Aires, Argentina.; 3 Rio de Janeiro RJ Brasil Departamento de Pediatria, Instituto D’Or de Pesquisa e Ensino - Rio de Janeiro (RJ), Brasil.

As unidades de terapia intensiva (UTIs) pediátricas concentram recursos humanos especializados e tecnologia avançada que podem melhorar o prognóstico de crianças em estado crítico. No entanto, embora a taxa de mortalidade em UTIs pediátricas tenha diminuído para aproximadamente 2% em países desenvolvidos, ela permanece próxima de 4% em outros, como os da América Latina. Portanto, é necessário avaliar o desempenho da UTI pediátrica e implementar estratégias de melhoria.^(1,2)^

A taxa de mortalidade padronizada, calculada como a razão entre as mortes observadas e as esperadas, é um indicador-chave para avaliar o desempenho de uma UTI pediátrica. Embora seja fácil avaliar a mortalidade observada, determinar a mortalidade esperada é um desafio. Os índices de gravidade da doença (SIS *- severity of illness scores*) surgiram como ferramentas que permitem estimar o risco de morte no momento da admissão. Além disso, os escores de disfunção orgânica (DO) foram desenvolvidos para descrever a evolução clínica de pacientes pediátricos em UTI.

Os SIS e os escores de DO podem ser usados para avaliar o desempenho institucional para fins de *benchmarking* interno e externo, orientando iniciativas de melhoria da qualidade, alocação de tratamento e pesquisa.^(3)^ Esse ponto de vista tem como objetivo fornecer um guia atualizado para aprimorar seu uso em ambientes de UTI pediátrica.

## ENTENDENDO OS ESCORES DE GRAVIDADE DA DOENÇA

A gravidade da doença é geralmente avaliada a partir de uma variedade de variáveis demográficas, clínicas, fisiológicas e laboratoriais. Os SISs usados em UTIs pediátricas baseiam-se na suposição de que há uma relação previsível entre a gravidade da doença no momento da admissão e o risco de morte na UTI pediátrica. Essencialmente, eles são modelos matemáticos derivados da aplicação de regressão logística passo a passo a uma coorte observacional, na qual um valor específico é atribuído a cada variável preditiva de mortalidade, resultando em chances finais transformadas em probabilidade de morte. As variáveis preditivas podem incluir alterações fisiológicas (como pressão arterial, frequência cardíaca e valores laboratoriais), a necessidade de intervenções (como ventilação mecânica e inotrópicos), a condição subjacente do paciente (como doença maligna) e a admissão de emergência na UTI pediátrica, entre outras. O desempenho do modelo resultante é, então, avaliado conforme sua discriminação e calibração gerais. Se ambas as medidas forem adequadas, antes de o modelo ser usado na prática de rotina, ele deverá ser validado em uma amostra separada de pacientes da mesma população (validação interna).

Considerando a heterogeneidade das populações nas UTIs pediátricas (idade, perfil de pacientes), os escores prognósticos são estabelecidos independentemente do diagnóstico para aumentar a objetividade da avaliação dos desfechos entre as instituições.

A descrição e a quantificação da DO durante a hospitalização na UTI pediátrica são importantes. A maior instabilidade fisiológica na admissão, secundária a uma doença mais grave, está correlacionada a um maior risco de desenvolver falência múltipla e sequencial de órgãos, o que aumenta a taxa de mortalidade. Os escores de DO foram desenvolvidos e validados para permitir a descrição do curso clínico e da gravidade da doença durante a internação na UTI pediátrica, não apenas para prever a mortalidade.

Os dois escores mais comumente usados para a previsão de mortalidade são as versões mais recentes do *Pediatric Risk of Mortality* (PRISM) e do *Pediatric Index of Mortality* (PIM).^(4,5)^ O *Pediatric Logistic Organ Disfunction Score* (PELOD) e o *Pediatric Sequential Organ Failure Assessment Score* (pSOFA) são usados para avaliar a disfunção de múltiplos órgãos.^(6,7)^ A tabela 1 apresenta esses escores, suas diretrizes de construção e suas limitações.

**Tabela 1:** Escores de mortalidade e gravidade da doença na unidade de terapia intensiva pediátrica

**PIM3**	Desenvolvido na Austrália, Nova Zelândia, Reino Unido e Irlanda^(5)^ **Versões anteriores:** PIM2 (2003); PIM (1997) **Variáveis (coletadas na primeira hora de admissão na UTI pediátrica):** reflexo pupilar | admissão eletiva ou de emergência | ventilação mecânica em qualquer momento durante a primeira hora | excesso de base | PAS | PaO_2_ e FiO_2_ | pós-operatório de cirurgia cardíaca com revascularização | pós-operatório de procedimento cardíaco sem revascularização | pós-operatório de cirurgia não cardíaca | motivo da admissão na UTI pediátrica (diagnóstico de risco muito alto: parada cardíaca antes da admissão na UTI, imunodeficiência combinada grave, leucemia ou linfoma após a primeira indução, receptor de transplante de medula óssea, insuficiência hepática; diagnóstico de alto risco: hemorragia cerebral espontânea, cardiomiopatia ou miocardite, síndrome do coração esquerdo hipoplásico, distúrbio neurodegenerativo, enterocolite necrosante; ou diagnóstico de baixo risco: asma, bronquiolite, crupe, apneia obstrutiva do sono, cetoacidose diabética, distúrbio convulsivo) **Cálculo:** Logit(P) = − 1,7928 + (3,8233×reflexo pupilar) − (0,5378×admissão eletiva) + (0,9763×ventilação mecânica) + (0,0671×(excesso de base absoluto)) − (0,0431×PAS) + (0,1716×(PAS×PAS/1.000)) + (0,4214×(100×FiO_2_/PaO_2_)) − (1,2246×pós-operatório de cirurgia cardíaca com revascularização) − (0,8762×pós-operatório de cirurgia cardíaca sem revascularização) − (1,5164×pós-operatório de cirurgia não cardíaca) + (1,6225×diagnóstico de risco muito alto) + (1,0725×diagnóstico de risco alto) − (2,1766×diagnóstico de baixo risco) **Probabilidade de morte:** P = exp(logit(P))÷(1 + exp(logit(P)) **Considerações especiais:** calcula a mortalidade na UTI pediátrica. A probabilidade de morte não é aplicável ao nível do paciente e o escore não pode ser usado para uma decisão diagnóstica ou terapêutica individual no manejo do paciente
**PRISM IV**	Desenvolvido nos Estados Unidos^(4)^ **Versões anteriores:** PRISM III (1996); PRISM II (1988); PSI (1984) Variáveis (para as variáveis laboratoriais, considerar os dados coletados entre as 2 horas anteriores à admissão e as primeiras 4 horas de admissão na UTI pediátrica e para as outras variáveis fisiológicas considerar os dados coletados nas primeiras 4 horas de atendimento na UTI pediátrica):* idade | origem da admissão (outro hospital, unidade de internação, emergência) | RCP nas 24 horas anteriores à admissão na UTI pediátrica | câncer (agudo ou crônico) | sistemas de baixo risco de disfunção primária (sistemas endócrino, hematológico, musculoesquelético e renal) | temperatura | escore neurológico: reatividade pupilar e estado mental (escala de coma de Glasgow) | escore não neurológico: frequência cardíaca, pressão arterial sistólica, temperatura, PaO_2_ arterial, pH ou CO_2_ total, PaCO_2_, bicarbonato total, glicose, potássio, nitrogênio ureico no sangue, creatinina, contagem de leucócitos, contagem de plaquetas, tempo de protrombina ou tromboplastina parcial **Cálculo:** Logit(P) = − 5,776 + (1,311×idade 0-14 dias) OU (0,968×idade 14 dias-1 mês) OU (0,357×idade 1-12 meses) + (1,012×admissão oriunda de outro hospital) OU (1,626×admissão oriunda da unidade de internação) OU (0,693×admissão oriunda da emergência) + (1,082×RCP nas 24 horas anteriores à admissão) + (0,766×câncer) − (1,697× sistemas de baixo risco) + (0,197×escore neurológico) + (0,163×escore não neurológico) *Se a admissão for para cirurgia cardíaca ou cateterismo cardíaco intervencionista (não diagnóstico), realizar o PRISM IV em até 4 horas após a cirurgia ou intervenção se o paciente tiver entre 12 horas e 10 dias de vida ou tiver entre 11 e 90 dias de vida e < 48 horas após a admissão na UTI pediátrica. **Probabilidade de morte:** P = exp(logit(P))÷(1 + exp(logit(P)) **Considerações especiais:** calcula a mortalidade hospitalar, o que pode afetar as comparações ao avaliar os resultados com outros escores. A probabilidade de morte não é aplicável ao nível do paciente, e o escore não pode ser usado para uma decisão diagnóstica ou terapêutica individual no manejo do paciente
**PELOD2**	Desenvolvido na França e na Bélgica^(6)^ **Versões anteriores:** PELOD (1999) **Variáveis (coletadas nas primeiras 24 horas e repetidas diariamente):** cardiovasculares (lactatemia e pressão arterial média) | respiratórias (PaO_2_/FiO_2_, PaCO_2_ e ventilação mecânica invasiva) | neurológicas (escala de coma de Glasgow e reatividade pupilar) | hematológicas (contagem de leucócitos e plaquetas) | renais (creatinina sérica) **Cálculo:** escore PELOD-2 = disfunção neurológica [(escala de coma de Glasgow: 0, 1 ou 4 pontos) + (reatividade pupilar: 0 ou 5 pontos)] + disfunção cardiovascular [(lactatemia: 0, 1 ou 4 pontos) + (pressão arterial média: 0, 2, 3 ou 6 pontos)] + disfunção renal (creatinina: 0 ou 2 pontos) + disfunção respiratória [(PaO_2_/FiO_2_: 0 ou 2 pontos) + (PaCO_2_: 0, 1 ou 3 pontos) + (ventilação invasiva: 0 ou 3 pontos)] + disfunção hematológica [(contagem de leucócitos: 0 ou 2 pontos) + (plaquetas: 0, 1 ou 2 pontos)] Logit(P) = - 6,61 + (0,47×escore PELOD-2) **Probabilidade de morte:** P = exp(logit(P))÷(1 + exp(logit(P)) **Considerações especiais:** os dados registrados pelo escore são influenciados pelo tratamento. O escore não pode diferenciar entre o tratamento incorreto e a gravidade da doença. As variáveis são medidas somente se o quadro clínico do paciente justificar, assumindo-se normalidade em caso contrário. A avaliação do estado neurológico é difícil devido à necessidade de sedação em pacientes graves, portanto, presume-se o estado de presedação.
**pSOFA**	Desenvolvido nos Estados Unidos a partir do SOFA para adultos usando critérios ajustados à idade^(7)^ **Versões anteriores:** SOFA (1996, versão para adultos) e Sepsis-3 (2016, versão para adultos) **Variáveis (coletadas diariamente durante a internação na UTI pediátrica):** respiratórias (PaO_2_/FiO_2_ OU SpO_2_/FiO_2_) | cardiovasculares (pressão arterial média OU infusão vasoativa) | hepáticas (nível sérico de bilirrubina) | coagulação (contagem de plaquetas) | renais (creatinina sérica) | neurológicas (escala de coma de Glasgow) **Cálculo:** pontuação pSOFA = respiratório [(PaO_2_/FiO_2_ OU SpO_2_/FiO_2_) + coagulação (contagem de plaquetas) + hepático (bilirrubina) + cardiovascular (pressão arterial média por faixa etária ou infusão de vasoativo) + neurológico (escala de coma de Glasgow) + renal (creatinina) pSOFA diário = o escore de cada 1 dos 6 sistemas varia de 0 a 4 pontos, o escore total varia de 0 a 24 pontos **Probabilidade de morte:** o escore pSOFA não foi validado para calcular o risco de morte. **Considerações especiais:** os dados registrados pelo escore são influenciados pelo tratamento. O escore não pode diferenciar entre o tratamento incorreto e a gravidade da doença. As variáveis são medidas somente se o quadro clínico do paciente justificar, assumindo-se normalidade em caso contrário. A avaliação do estado neurológico é difícil devido à necessidade de sedação em pacientes graves, portanto, presume-se o estado de presedação.

PIM - *Pediatric Index of Mortality;* UTI - unidade de terapia intensiva; PAS - pressão arterial sistólica; PaO_2_ - pressão parcial de oxigênio; FiO_2_ - fração inspirada de oxigênio; PRISM - *Pediatric Risk of Mortality*; RCP - reanimação cardiopulmonar; CO_2_ - gás carbônico; PaCO_2_ - pressão parcial de gás carbônico; PELOD - *Pediatric Logistic Organ Disfunction Score*; SOFA - *Sequential Organ Failure Assessment*.

## PONTOS-CHAVE DA PRÁTICA CLÍNICA

A seleção de um SIS ou DO pressupõe a consideração de vários fatores, como garantir que ele tenha sido validado localmente, que seja atualizado e que o pessoal responsável pela entrada de dados seja treinado nas diretrizes de construção do escore para garantir que os dados sejam de alta qualidade.

Em geral, os escores de gravidade são desenvolvidos em países de alta renda. Caso se pretenda usar um SIS em populações diferentes daquela para a qual foi desenvolvido, ele deverá ser validado externamente por meio da realização de testes de discriminação e calibração. A discriminação é uma medida da capacidade de um escore de atribuir probabilidades menores de morte a pacientes que viverão e probabilidades maiores de morte àqueles que morrerão. Ela é avaliada calculando-se a área sob a curva *Receiver Operating Characteristic* (ROC). Uma área sob a curva ROC igual a 0,50 significa que a pontuação não é mais discriminatória do que o acaso, uma área entre 0,70 e 0,79 é considerada adequada, uma área entre 0,80 e 0,89 é considerada boa e uma área > 0,90 é considerada excelente.

A calibração é uma medida de quão bem a mortalidade prevista corresponde à mortalidade observada por nível de gravidade no momento da admissão na UTI pediátrica. Testes estatísticos específicos (por exemplo: Hosmer-Lemeshow e cinturões de calibração) são usados para comparar as mortes observadas e previstas por faixa de gravidade.^(8-10)^ Quando a calibração de um escore em uma população é boa, o número de mortes observadas em cada decil de gravidade é semelhante ao número de mortes previstas pelo escore na mesma faixa de risco. A validação externa dos escores de previsão de mortalidade geralmente indica uma calibração ruim. Em três grandes estudos recentes realizados na América Latina, o PIM2 e o PIM3 apresentaram boa discriminação, mas calibração ruim em vários subgrupos de pacientes.^(11-13)^ Esse achado deve ser interpretado com cautela. Embora possa ser devido aos diferentes desempenhos das UTIs pediátricas locais, o resultado pode ter sido influenciado por variações regionais ou nacionais na organização da assistência e diferentes combinações de pacientes e casos. Nesse caso, seria útil validar o escore em uma amostra representativa local e considerar a taxa de mortalidade padronizada obtida como 1 (um) nessa população.

À medida que os tratamentos de cuidados intensivos melhoram, os desfechos para a mesma gravidade de doença podem mudar. Portanto, é necessário atualizar um SIS recalculando-se o coeficiente de cada variável ou incorporando novas variáveis. Uma avaliação precisa do desempenho da UTI pediátrica exige o uso de escores atualizados.

A qualidade dos dados é uma consideração importante ao se desenvolver e usar um escore. Erros no registro de dados e dados ausentes podem distorcer os resultados até mesmo do escore mais sofisticado. Portanto, é fundamental entender as regras de construção dos escores que serão usados na UTI pediátrica.

Por fim, embora as probabilidades de mortalidade de pacientes específicos possam ser calculadas, elas não devem orientar as decisões relativas à limitação do suporte à vida.

## CONCLUSÃO

As unidades de terapia intensiva pediátricas devem usar índices de gravidade da doença atualizados e, de preferência, validados localmente para medir seu desempenho. Além disso, medir o grau de disfunção orgânica no momento da admissão e durante a internação na unidade de terapia intensiva pediátrica permite avaliar a evolução de um paciente e pode ser usado como critério de inclusão e exclusão de protocolos de tratamento. A implementação de procedimentos para garantir a qualidade dos dados, como a coleta de dados em tempo real por pessoal adequadamente treinado, é muito importante para garantir que as estimativas sejam confiáveis.
